# Mechanism of anti-remodelling action of treprostinil in human pulmonary arterial smooth muscle cells

**DOI:** 10.1371/journal.pone.0205195

**Published:** 2018-11-01

**Authors:** Christopher Lambers, Christoph Kornauth, Felicitas Oberndorfer, Panja M. Boehm, Michael Tamm, Walter Klepetko, Michael Roth

**Affiliations:** 1 Thoracic Surgery, University Hospital Vienna, Vienna, Austria; 2 Clinical Institute of Pathology, Medical University of Vienna, Vienna, Austria; 3 Pulmonary Cell Research and Pneumology Clinics, University and University Hospital Basel, Basel, Switzerland; Qatar University College of Health Sciences, QATAR

## Abstract

Treprostinil is applied for pulmonary arterial hypertension (PAH) therapy. However, the mechanism by which the drug achieves its beneficial effects in PAH vessels is not fully understood. This study investigated the effects of treprostinil on PDGF-BB induced remodelling parameters in isolated human pulmonary arterial smooth muscle cells (PASMC) of four PAH patients. The production of TGF-β1, CTGF, collagen type-I and -IV, and of fibronectin were determined by ELISA and PCR. The role of cAMP was determined by ELISA and di-deoxyadenosine treatment. Proliferation was determined by direct cell count. Treprostinil increased cAMP levels dose and time dependently, which was not affected by PDGF-BB. Treprostinil significantly reduced PDGF-BB induced secretion of TGF-β1 and CTGF, both was counteracted when cAMP generation was blocked. Similarly, the PDGF-BB induced proliferation of PASMC was dose dependently reduced by treprostinil through signalling via cAMP—C/EBP-α p42 –p21^(WAf1/Cip1)^. In regards to extracellular matrix remodelling, treprostinil significantly reduced PDGF-BB—TGF-β1—CTGF induced synthesis and deposition of collagen type I and fibronectin, in a cAMP sensitive manner. In contrast, the deposition of collagen IV was not affected. The data suggest that this action of treprostinil in vessel wall remodelling may benefit patients with PAH and may reduce arterial wall remodelling.

## Introduction

Pulmonary arterial hypertension (PAH) is a devastating disease with limited therapeutic options [1]. PAH is defined by progressive increase of vascular resistance in pulmonary blood vessels, which is assumed to increase the blood pressure in the lung locally. In long term, this leads to right heart failure and premature death [2]. Monotherapy with prostacyclin analogues improved long term survival rates and function significantly, while the effects of combination therapies are under investigation [3]. The initiation of PAH is still unknown and several mechanisms have been suggested, including local over-activity of calcium sensing receptors, G-proteins and platelet derived growth factor (PDGF)-BB, as well as endothelin-1 and transforming growth factor (TGF)-β [4–7].

The best option for slowing the progression of the disease is today the use of prostacyclin analogues, which improve hemodynamic function and survival. Prostacyclin analogues have been tested in clinical studies to be safe and result in less side effect than other drugs [8–10]. Inhaled prostacyclin analogues had recently been considered to replace infusion but have to be applied several times within 24 hours. Inhalation of drugs such as treprostinil at high doses in PAH therapy have been recently proven as safe and reasonably well tolerated [11]. However, there is contradicting data in regards to long term benefits of inhaled prostacyclin analogues when compared to oral or infusion application [10, 12].

Treprostinil is a stable prostacyclin analogue, which achieves vasodilation of the pulmonary muscle and it reduces platelet aggregation. In animal models, treprostinil increased the activation of PPAR-γ, which resulted in a reduced proliferation rate of muscle cells [13]. This effect as well as the reduced activity of human erythrocytes, reduced adhesion and differentiation of fibrocytes was linked to increased cAMP synthesis in the different cell types [14, 15]. However, there is little knowledge on the effects of treprostinil on growth factors that are associated with the pathogenesis of PAH such as PDGF-BB and TGF-β. In PAH, an over active TGF-β1 signalling cascade has been described which was also linked to the increased proliferation of PASMC in an animal model. In a study including 44 patients with idiopathic inherited PAH, the baseline plasma levels of 4 factors, including PDGF-BB were increased compared to controls. However, treatment with treprostinil improved clinical parameters but did not affect any of the growth factors [16]. The inhibition of the pathway by treprostinil reduced the cell proliferation [17].

Recently, precursor and break down products of collagens are discussed as biomarkers for the progression and staging of PAH [18, 19]. In regards to the above described association of PDGF-BB with PAH vessel remodelling, it is important to note that the inhibition of PDGF-BB reduced PASMC remodelling and collagen deposition in a model for PAH [20]. Most studies in regard of vessel wall remodelling in PAH only focussed on the proliferation of PASMC and did not investigate the effect of the drugs on other parameters for remodelling, such as increased deposition of extracellular matrix or of its pro-inflammatory components collagen type I and fibronectin. In other conditions, we reported earlier that cAMP signalling was involved in extracellular matrix metabolism as well as in proliferation control [21, 22]. Furthermore, we were the first to link cAMP signalling to the negative cell cycle regulator p21^(Waf1/Cip1)^ in human airway cells [23]. The expression of p21^(Waf1/Cip1)^ is regulated by C/EBP-α, which in turn is controlled by cAMP [24–26]. Thus, it is likely that treprostinil controls proliferation through a signalling cascade that involves: cAMP—C/EBP-α—p21^(Waf/Cip1)^, while collagen synthesis and deposition is controlled through cAMP—TGF-β1.

In this study, we investigated the effect of treprostinil on cAMP activation and on PDGF-BB induced remodelling parameters, which included the secretion of TGF-β1, connective tissue growth factor (CTGF), PASMC proliferation and deposition of collagen type I, collagen type IV and fibronectin. All experiments were performed in isolated primary PASMC.

## Materials and methods

### PASMC

PASMC from four explanted lungs obtained from patients suffering from PAH were isolated and characterised as described earlier [27]. The details of the patients are provided in Table 1.

**Table 1:** PAH patient demographics.

Age (years)	Sex	BMI	Underlying disease	sPAP (Echo)	Therapy
34	female	22,95	PPH	100 mmHg	Sildenafil, Volibris, Remodulin
48	male	15,64	PPH, LMCA	80 mmHg	Revatio, Opsumit
39	male	20,11	PPH	125 mmHg	Remodulin
26	male	24,21	PPH	85 mmHg	Remodulin, Opsumit

Abbreviations: BMI = body mass index, PPH = primary pulmonary hypertension; LMCA = compression of the left main artery; sPAP = systolic pulmonary artery pressure

CC-2581 cells representing non-diseased primary human PASMC (Cambrex Bioscience, Walkersville, USA) were used as control. The growth medium was: DMEM supplemented with 5% foetal calf serum, 20 mM HEPES, 8 mM L-glutamine and 1 x antibiotic/anti-mycotic mixture (all Gibco/BRL, Vienna, Austria).

All experiments were performed in sub-confluent (80%) PASMC. Cells were characterised by the expression of α-smooth muscle actin. All experiments were performed between passages 3–6 [27].

### Treatment

PASMC were serum deprived for 24 hours before stimulation with human recombinant PDGF-BB (1–10 ng/ml; R&D Systems, Abington, UK) for 20 and 60 minutes (cAMP), 24 hours for collagens and fibronectin and 48 hours for proliferation.

To assess the effect of Treprostinil, PASMC were incubated for 60 minutes prior to PDGF-BB stimulation with increasing concentration of the drug (10^−6^, 10^−8^, 10^−10^ M).

### Cyclic AMP (cAMP), TGF-β1 and CTGF ELISA

Serum deprived PASMC were treated with Treprostinil (10^−10^–10^−6^ M), or forskolin (10μM), and/or, 100μM) for 0, 1, 3, or 6 hours. To inhibit cAMP increase cells were pre-treated for 30 minutes with 10 μM DDA. Cells were lysed and intracellular cAMP was determined by a commercial ELISA kit, following the producer’s instructions (Assay Designs Inc., Ann Arbor, USA).

The secretion of TGF-β and CTGF were determined in cell culture medium collected after 24 hours by commercial ELISAs (R&D Systems, Abington, UK) according to the instruction of the distributor.

### TGF-β and CTGF

Real Time (RT)-PCR was performed using a LightCycler480 (Roche Diagnostics, Mannheim, Germany) for mRNA encoding: connective tissue growth factor (CTGF), TGF-β_1_, pro-collagen-1A1 (Col-1A1) and fibronectin (TaqMan gene expression assays, Applied Biosystems, CA, USA). RT-PCR conditions: 5 min denaturing (95°C), followed by 45 cycles: 10 seconds denaturing at 95°C; 30 seconds annealing at 65°C; 5 seconds elongation at 72°C. Human 18S-RNA or actin were used as controls (TaqMan Pre-Developed Assay, Applied Biosystems).

### Extracellular matrix deposition

The deposition of collagen type-I and -IV and fibronectin was determined by a cell based in-house developed ELISA as described earlier [21] All antibodies used for the ELISA were purchased from Santa Cruz Bio Technology, Santa Cruz, USA (Col-1A1 sc-8784, Col-4A1 sc-385020, fibronectin sc-6952). Secondary HRP and fluorescence labelled antibodies were selected according to the species of the first antibodies and purchased from Santa Cruz Bio Technology.

### Cell proliferation

Cell numbers were counted directly after seeding and adjusted to 10,000 cells per cm^2^ and 48 hours after the start of stimulation. Cells were trypsinized and cell numbers were counted manually using an improved Neugebaur Chamber slide.

### Immunoblot analysis

Proteins (25 μg) were size fractionated by gel electrophoresis (12% SDS-polyacrylamide gel), and transferred onto a PVDF membrane (Millipore Corp., Billerica, USA) by standard protocol. Protein transfer was confirmed by Ponceau red staining. Membranes were incubated (2 hours, room temperature: RT) in blocking buffer (PBS, 0.1% Tween-20, 5% skimmed milk) before incubation with a first monoclonal antibody overnight (4°C) [22]. The membranes were washed 3x (80 nM Na_2_HPO_4_, 20 mM NaH_2_PO_4_, 100 mM NaCl, 0.1% Tween-20, pH 7.5) and then incubated (90 min., RT) with a secondary horse radish peroxidase-labelled species specific antibody (Amersham, Buckinghamshire, UK). After three washes with PBS protein bands were visualized by enhanced chemiluminescence substrate (Pierce, Illinois, USA) and documented on X-ray film for image analysis (ImageJ version 1.37). Antibodies used: actin (A-2066, Sigma), p21^(Waf1/Cip1)^ (AB3003, Chemicon) C/EBP-α (sc-9314, Santa Cruz) and C/EBP-β (sc-2962, Santa Cruz).

### Statistics

Two sets of analyses were conducted. Firstly, in the “n = 3” analyses the Null hypotheses was: “no difference comparing the mean of: A vs B, B vs C, C vs D. The sample size in each group was n = 3 and observations were paired, since the data originated from the same cell line. For this analysis paired t-tests was applied. All n = 3 analyses are graphically represented as dot-plots with mean, and only significant differences between groups are shown in the figures. The second analysis was applied to experiments with “n = 6” and assessed the Null hypothesis: “no difference comparing the mean between groups: “Control”, “PDGF”, “PDGF+Trep10^-6^ M” and”PDGF+Trep10^-8^ M”.

Furthermore, the pairwise comparisons were made for “Control vs PDGF”, “PDGF vs PDGF+Trep10^-6^ M” and “PDGF vs. Trep10^-8^ M”. The sample size in each group was n = 6 and the observations were independent, as they were the result of separate experiments. The Null hypotheses were tested using ANOVA and pairwise comparisons were conducted. All “n = 6” analyses are graphically represented as mean+-se bars. Only significant differences between groups, as well as the overall ANOVA p-values are shown in the figures.

For both the “n = 3” and “n = 6” analyses, the p-value tables of all conducted statistic analyses are reported in the S1 Appendix. Since this is an exploratory/proof-of-concept study and p-values serve only descriptive purposes, no multiplicity corrections were applied.

## Results

### PAH pathologies

**Fig 1:**
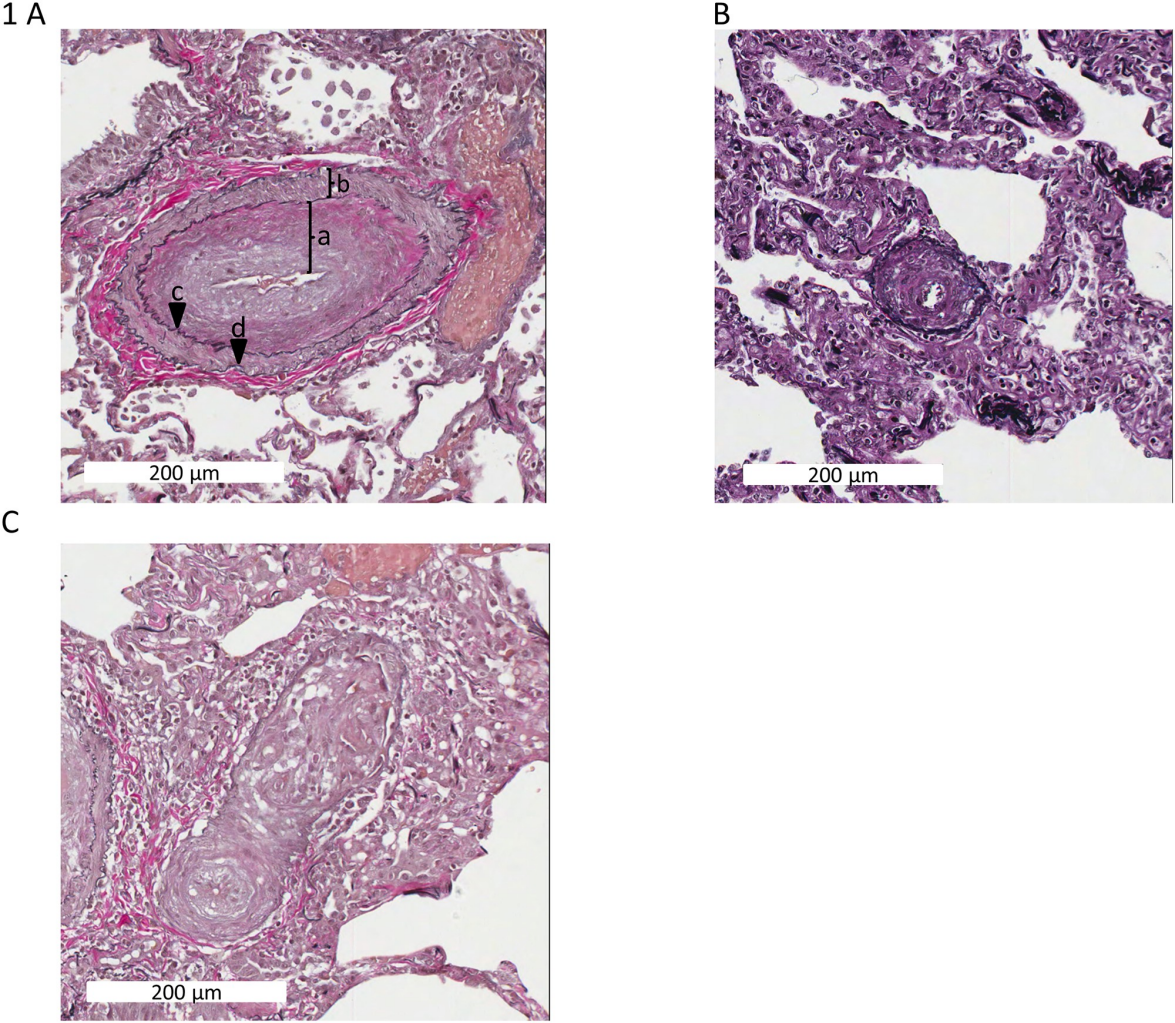
Characteristic histology of PAH vessel remodelling. (A) Fibroelastic intimal hyperplasia [a) Intima; b) Muscularis media; c) Elastic interna; d) Elastica externa]. (B) Concentric medial hyperplasia (C) Angiomatoid lesion; Elastica van Gieson; Scale bar 200μm in A and C, 100μm in B.

Representative histologies of the characteristic vascular pathology of pulmonary hypertension were obtained from patients (Fig 1). The most striking feature is remodelling of the vessel wall characterized by expansion of vessel wall components through deposition of extracellular matrix material and muscle hyperplasia. The intimal and medial layers are widened by fibroelastosis (consistent with expansion of extracellular matrix) and smooth muscle hyperplasia in respectively. In severe cases, angiomatoid/plexiform lesions are seen, which represent multiple vascular slits with endothelial lining surrounded by expanded myofibroblastic tissue.

### Treprostinil prevents PDGF-BB induced secretion of pro-remodelling factors through cAMP

**Fig 2:**
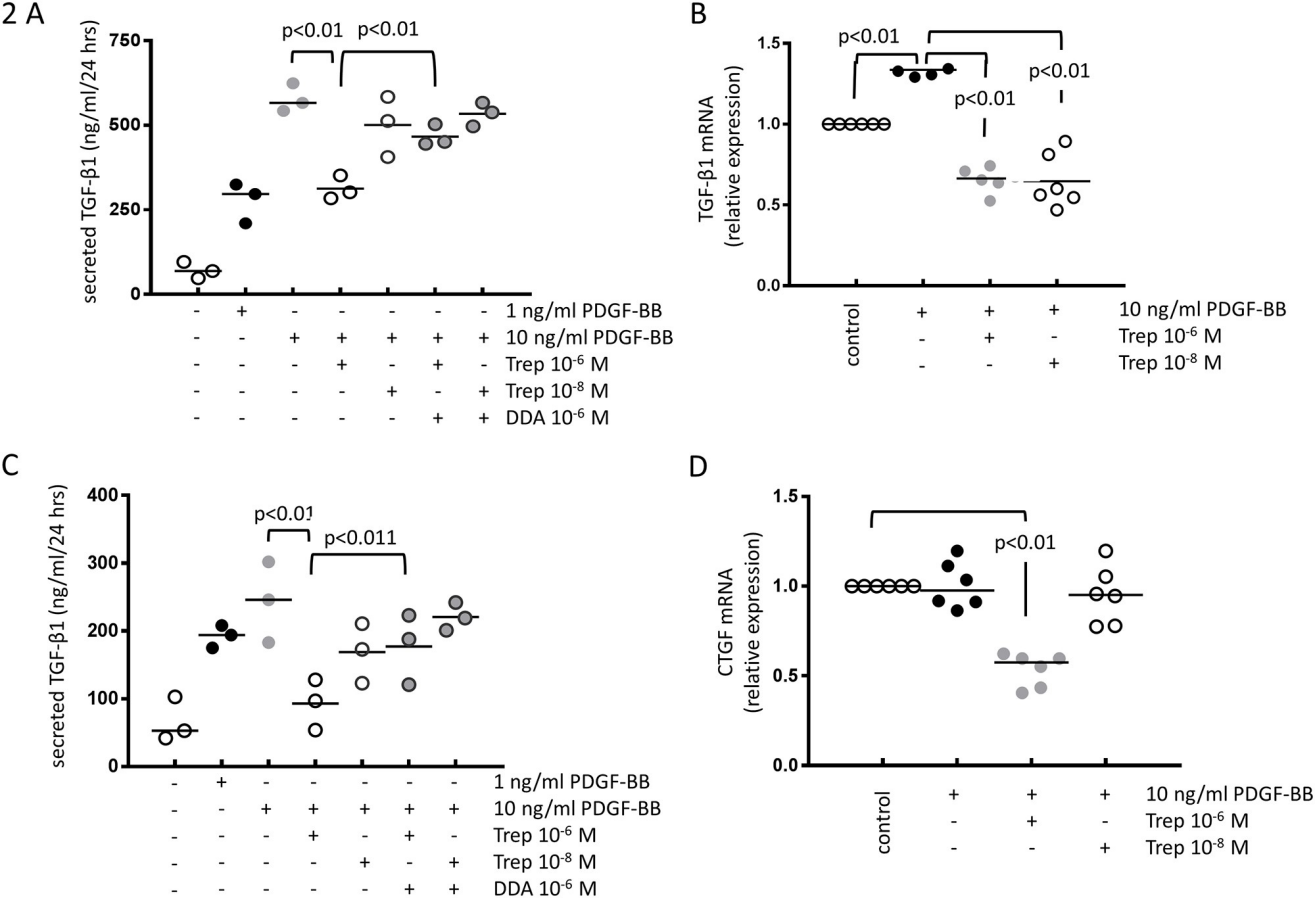
(A) The inhibitory effect of treprostinil on the *de novo* synthesis of TGF-β1 on the level of protein secretion (n = 3) and (B) mRNA synthesis (n = 6). The inhibitory effect of treprostinil on (C) CTGF secretion (n = 3) and (D) CTGF mRNA synthesis (n = 6).

In PASMC of four PAH patients, PDGF-BB (1–10 ng/ml) induced TGF-β1 secretion significantly, which was prevented by pre-incubation (30 min) of the cells with treprostinil (10^−8^–10^−6^ M) in a concentration dependent manner (Fig 2A). The inhibitory effect of treprostinil was significantly reduced in the presence of the cAMP blocker DDA (10^−6^ M), which was added 15 minutes prior to the drug (Fig 2A). The inhibitory effect of treprostinil on PDGF-BB induced TGF-β1 secretion was due to reduced transcription as shown by RT-PCR (Fig 2B).

Similarly, CTGF secretion was increased by PDGF-BB (Fig 2C). This effect was prevented in concentration dependent manner by pre-incubating the cells (30 min.) with treprostinil (Fig 2C). In the presence of DDA (10^−6^ M), the preventive effect of treprostinil did not occur (Fig 2C). The inhibitory effect of treprostinil on PDGF-BB induced CTGF expression was confirmed on the level of transcription (Fig 2D). Similar effects were obtained in the control cell line, which showed no difference when compared to the PAH cells (data not shown).

**Fig 3:**
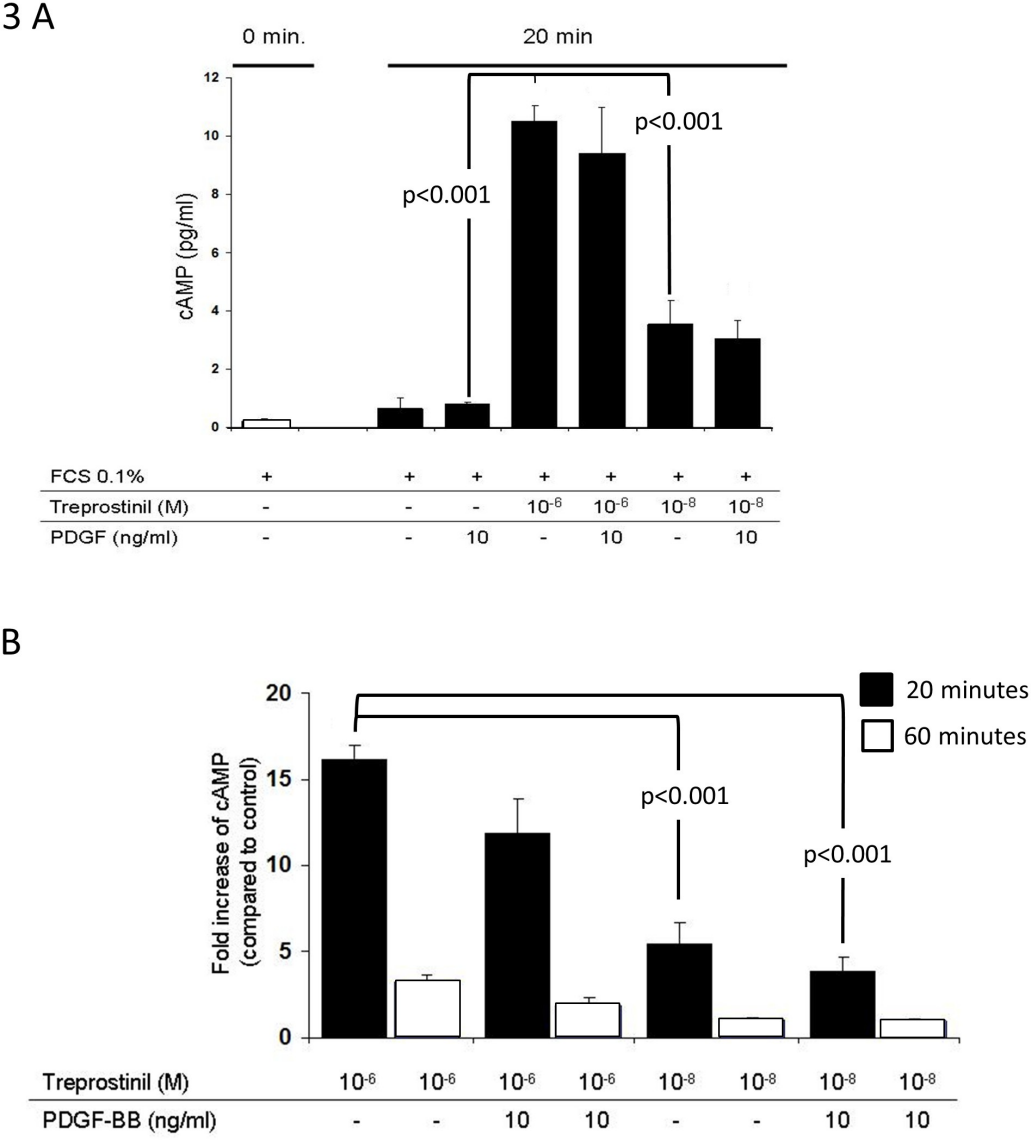
The effect of treprostinil on intracellular cAMP generation and its modification by PDGF-BB. (A) at 20 minutes and (B) at 20 versus 60 minutes (both n = 3).

In PAH derived PASMC as well as in the control cell line, PDGF-BB had no stimulating effect on intracellular cAMP (Fig 3A). Treprostinil (10^−6^, 10^−8^ M) significantly up-regulated cAMP within 20 minutes in a concentration dependent manner (Fig 3A). Treprostinil induced cAMP was reduced by PDGF-BB, but the effect did not achieve significance (Fig 3A). However, the reducing effect of PDGF-BB on treprostinil-induced cAMP was also detectable at a second time point (60 min.) and at 2 concentrations of the drug (Fig 3B).

### Treprostinil reduces extracellular matrix deposition with a component specific effect

**Fig 4:**
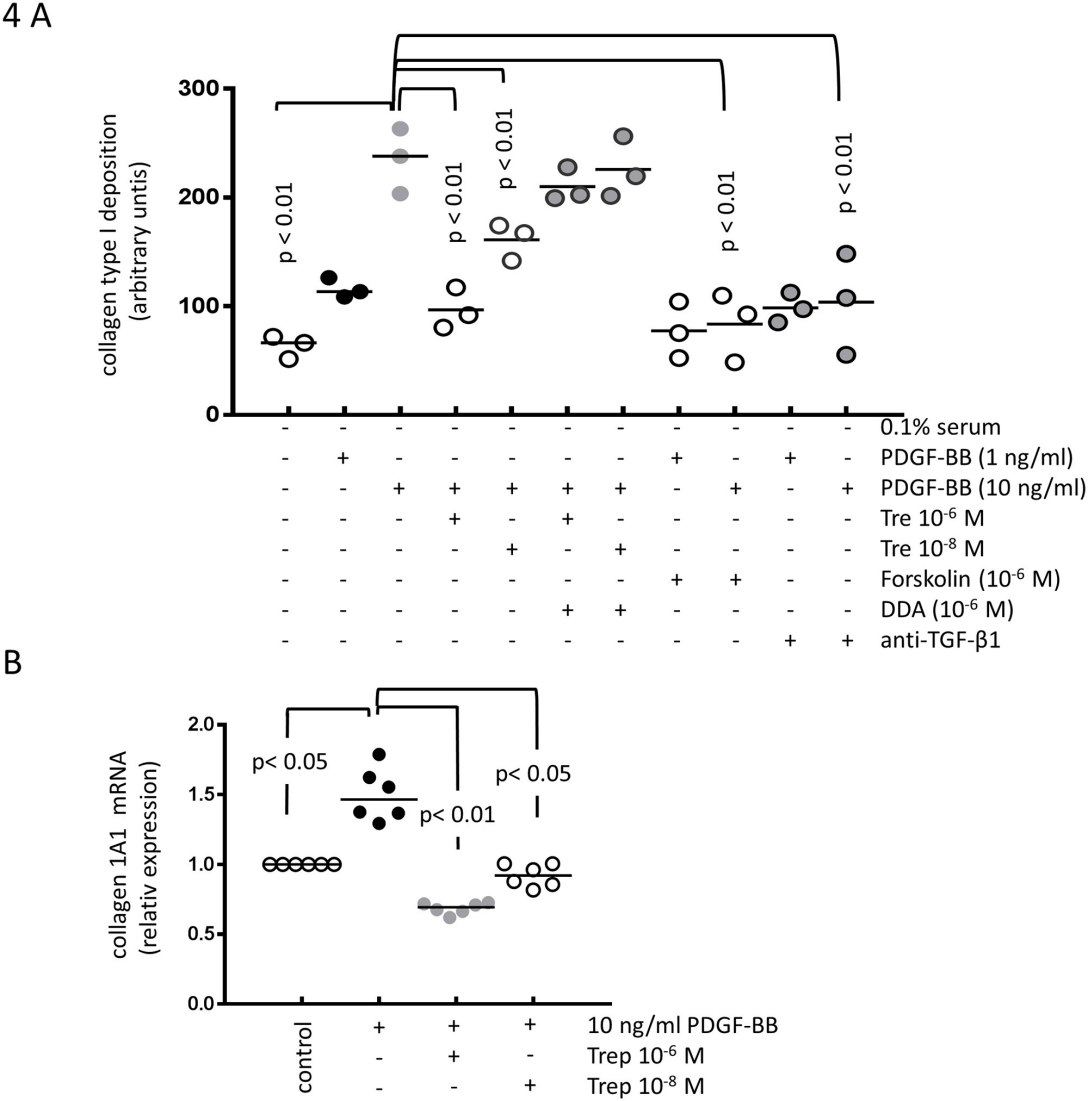
The inhibitory effect of treprostinil and the role of cAMP and TGF-β on PDGF-BB induced. (A) collagen type-I deposition (n = 3) and (B) *de novo* synthesis of COL1A1 mRNA (n = 6).

In primary PASMC obtained from four PAH patients, PDGF-BB (1–10 ng/ml) significantly increased the deposition of collagen type I and this effect was prevented by pre-incubation with treprostinil in a concentration dependent manner (Fig 4A). The presence of DDA significantly counteracted the reducing effect of treprostinil, suggesting that cAMP is a major signal mediator (Fig 4A). PDGF-BB induced collagen type-I deposition was also reduced by Forskolin (10^−6^ M) and by neutralising anti-TGF-β1 antibody (Fig 4A). The inhibitory effect of treprostinil on PDGF-BB induced collagen type-I synthesis and deposition was due to reduced transcription of the gene encoding collagen IA1 chain (Fig 4B). We did not observe any difference comparing the results obtained in PAH derived PASMC to the control cell line CC-2581.

In PAH PASMC and in CC-2581, PDGF-BB marginally up-regulated the deposition of collagen type IV. However, neither treprostinil nor DDA nor the anti-TGF-β1 antibody had any significant effect (data not shown).

**Fig 5:**
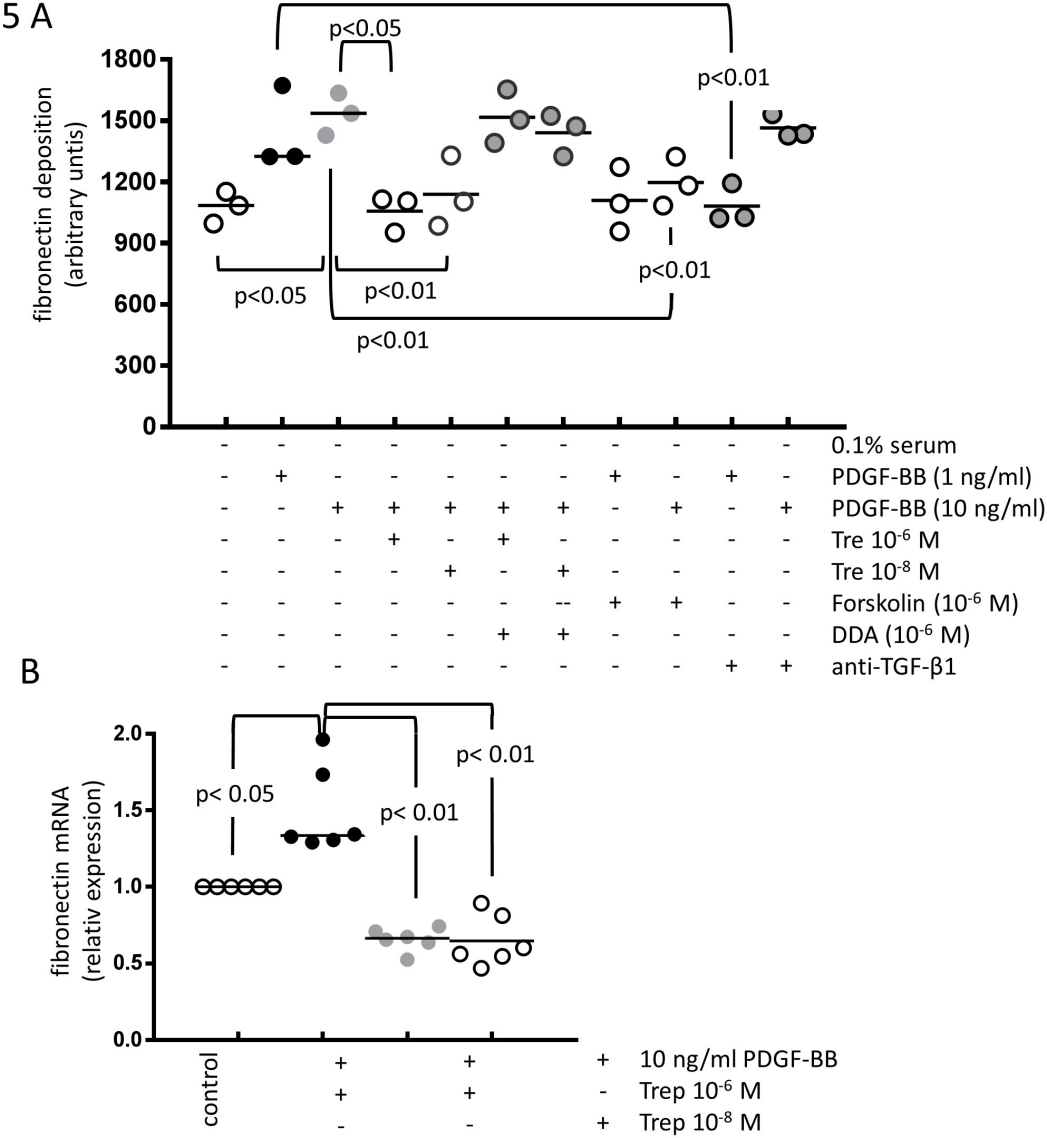
The inhibitory effect of treprostinil and the role of cAMP and TGF-β on PDGF-BB induced. (A) fibronectin deposition (n = 3) and (B) *de novo* synthesis of fibronectin mRNA (n = 6).

Treprostinil prevented PDGF-BB induced deposition of fibronectin (Fig 5A) by PAH derived PASMC. Pre-incubation of the cells with DDA reversed the effect of treprostinil (Fig 5A). PDGF-BB induced fibronectin deposition was reduced in the presence of Forskolin (10^−6^ M) but was only significantly reduced at the lower concentration of PDGF-BB (1 ng/ml) by the presence of anti-TGF-β1 antibody (Fig 5A). The reducing effect of treprostinil on PDGF-BB induced fibronectin deposition was due to reduced transcription of the fibronectin gene (Fig 5B). Similar results were obtained in CC-2581 cells (data not shown).

### Treprostinil inhibits PASMC proliferation by up-regulating C/EBP-α p42 and p21^(Waf1/Cip1)^

**Fig 6:**
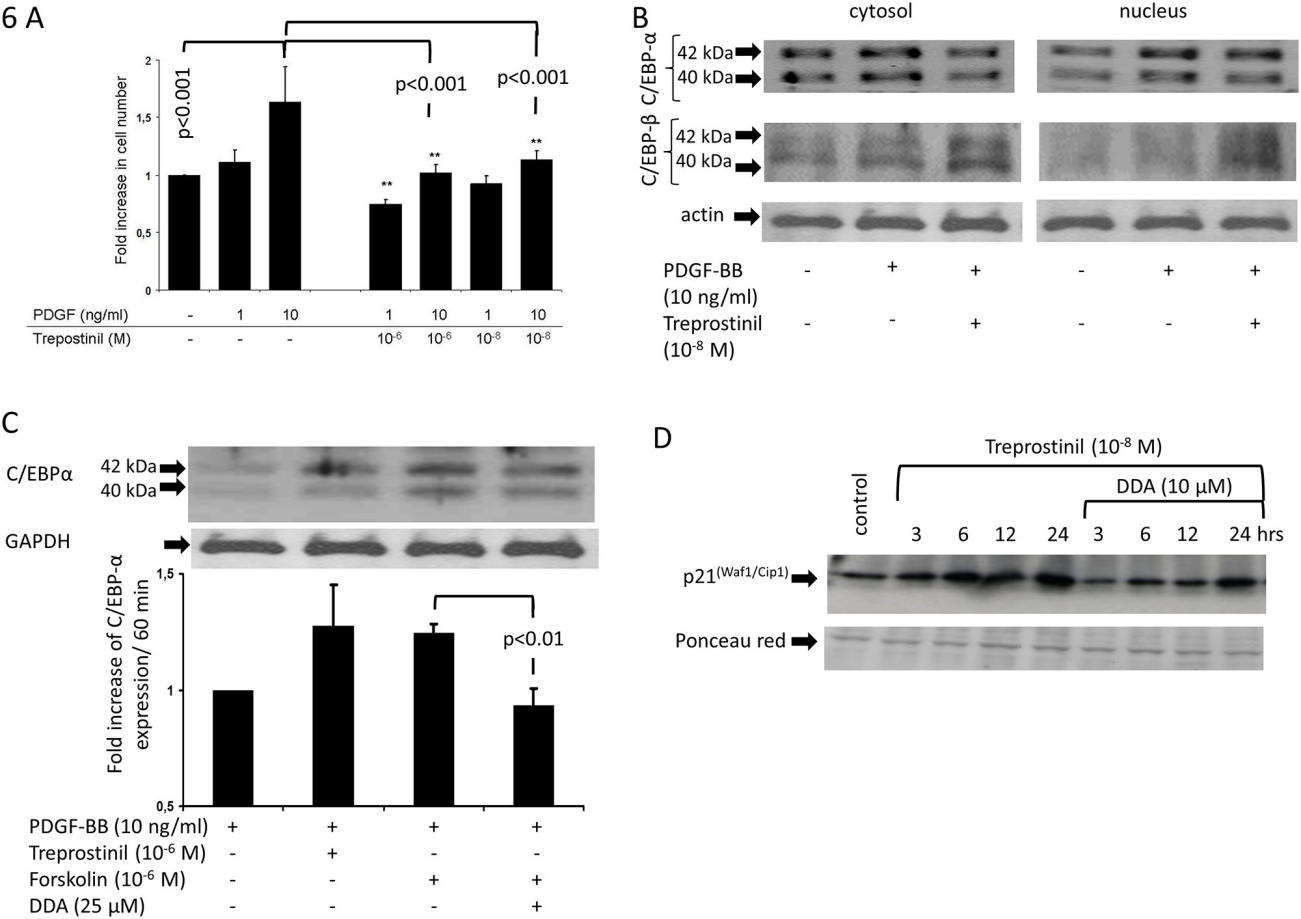
The role of C/EBP-α and C/EBP-β in treprostinil induced anti-proliferative p21^(Waf1/Cip1)^ signalling. (A) Treprostinil reduced PDGF-BB induced PASMC proliferation (n = 3). (B) Representative immune-blot of treprostinil induced activation (cytosol—nucleus transfer) of C/EBP-α and C/EBP-β in PASMC. Similar results were achieved in PASMC of two additional PAH patients. (C) Immuno-blot and graphical analysis of cAMP dependent suppression of C/EBP-α accumulation in the nucleus. (n = 3) (D) The effect of treporstinil and DDA on the expression of p21^(Waf1/Cip1)^ in PASMC.

Treprostinil dose dependently reduced PDGF-BB induced proliferation of PAH derived PASMC (Fig 6A). Compared to unstimulated control cells, PDGF-BB had no significant effect on the expression of C/EBP-α over the time course of 6 hours (Fig 6B). PDGF-BB significantly increased the expression of C/EBP-α p42 and pre-incubation of PASMC with treprostinil induced its translocation into the nucleus (Fig 6B). C/EBP-β expression was not significantly modified by PDGF-BB or treprostinil (Fig 6B). A similar stimulating effect on C/EBP-α was observed with forskolin which was reduced in cells that had been pre-incubated with DDA (Fig 6C).

Treprostinil activated the target of C/EBP-α, p21^(Waf1/Cip1)^ (Fig 6D). Pre-incubation with DDA reduced the stimulatory effect of treprostinil on p21^(Waf1/Cip1)^ (Fig 6D). Similar results, were obtained in CC-2581 cells, which were not different from PAH-PASMC (data not shown).

## Discussion

In this study, treprostinil significantly reduced or prevented parameters which indicate vessel wall remodelling. This beneficial effect of treprostinil depends on the generation of cAMP which inhibits PDGF-BB and sub-sequent TGF-β1 signalling. In consequence, this reduced the deposition of collagen type-I, and fibronectin. Furthermore, cAMP was linked to the anti-proliferative effect of treprostinil.

In PASC, treprostinil increased the level of intracellular cAMP within minutes and this was partly reduced by PDGF-BB. In line with these findings, treprostinil and other prostacyclin analogues achieved most of their effects by increasing cAMP. Different prostacyclin analogues have been reported with drugs specific anti-proliferative efficacy on serum stimulated PASMC proliferation, however, all drug effects were sensitive to cAMP [28]. In another study, proliferation of PAH patient’s PASMC was sensitive to prostacyclin analogues involving PPAR-γ signalling, but was insensitive to cAMP [29]. Nevertheless, phosphodiesterase inhibitors blocked cell activation through a signalling cascade that involved both PPAR-γ and cAMP [30]. Other data suggested a cooperative action of cAMP with cGMP signalling in a mouse model of bleomycin induced PAH [31]. The study suggested that the combination of prostacyclin analogues with phosphodiestrase-2 inhibitors has a synergistic anti-proliferative effect on PASMC. In inherited PAH, iloprost and treprostinil compensated for the loss of the bone morphogenic protein type-II receptor through a cAMP dependent inhibition of Smad6 [32]. The inhibition of Smad6 by cAMP rescued the phosphorylation of Smad1/5 in the bone morphogenic protein signalling cascade, thereby reducing the proliferation of PASMC in both healthy and PAH derived cells.

This is the first data showing that treprostinil prevented PDGF-BB induced PASMC proliferation, which was paralleled by an increase of the cell cycle inhibitors C/EBP-α and its target protein p21^(Waf1/Cip1)^. Both factors were essential to block cell cycle progression of airway smooth muscle cells and the lack of C/EBP-α p42 in asthma increased cell proliferation [24, 33]. In asthmatic smooth muscle cells only the largest isoform of C/EBP-α p42 was anti-proliferative, while all other isoforms (p40, p30, p22) acted as competitors of C/EBP-α p42 [34, 35]. Treprostinil specifically up-regulated the expression and nuclear accumulation of C/EBP-α p42, while the other isoforms were maintained at their base line expression. The treprostinil-induced increase of C/EBP-α p42 was followed by the up-regulation of p21^(Waf1/Cip1)^ expression, which is a potent inhibitor of the G0-S-phase transition of the cell cycle [33]. In contrast, treprostinil had no detectable effect on C/EBP-β expression. Prostaglandins activated C/EBP-α and PPAR-γ, which are essential for the differentiation of other vessel cell types to undergo differentiation [36]. Thus, treprostinil may increase PASMC differentiation.

Beside proliferation, the increased deposition of extracellular matrix components is a hallmark of PAH. Increased collagen type-I deposition in pulmonary arterial vessel walls has been demonstrated during remodelling in PAH both in humans and animals [37, 38]. PDGF-BB is a major stimulus for PASMC proliferation as well as for increased deposition of extracellular matrix components especially of pro-inflammatory collagens [20]. The above-described activation of PPAR-γ by prostacyclin analogues may also explain the beneficial effects of treprostinil on remodelling in PAH. This beneficial effect may also involve the interference of PPAR-γ with ERK-1/2 MAPK and cAMP signalling [39]. The latter observation may be linked to the earlier reported co-activation of PPAR-γ by treprostinil, which involved increased cAMP in PASMC [28]. Similar anti-remodelling mechanisms for iloprost, both anti-proliferative and anti-remodelling, have been described in experimental PAH [40]. However, the role of cAMP and vessel remodelling under PAH conditions has not been investigated in details.

### Conclusion

Activation of cAMP signalling by treprostinil may have beneficial preventive effects on pulmonary artery vessel wall remodelling by down-regulating proliferation and deposition of collagen I and fibronectin.

## Supporting information

S1 Appendix Statistical significant results for the presented data in Figs 2, 4 and 5. (DOCX)
