# Supplementary material for: Mechanism of anti-remodelling action of treprostinil in human pulmonary arterial smooth muscle cells
Source: PLoS One. 2018 Nov 1;13(11):e0205195. doi: 10.1371/journal.pone.0205195 (PMC6211661; doi:10.1371/journal.pone.0205195)
Supplement: S1 Appendix — (DOCX) [file pone.0205195.s001.docx]

Supplementary Table 1: Means and Standard Deviations of TGF-β1 expression in Figure 2B

|  | Control | PDGF | PDGF+Trep-6 | PDGF+Trep-8 |
| --- | --- | --- | --- | --- |
| Mean*1000 | 0.54 | 0.81 | 0.53 | 0.52 |
| Standard Deviation*1000 | 0.08 | 0.15 | 0.06 | 0.13 |

Supplementary Table 2: Means and Standard Deviations of CTGF expression in Figure 2D

|  | Control | PDGF | PDGF+Trep-6 | PDGF+Trep-8 |
| --- | --- | --- | --- | --- |
| Mean*1000 | 0.79 | 0.80 | 0.43 | 0.76 |
| Standard Deviation*1000 | 0.12 | 0.10 | 0.07 | 0.13 |

Supplementary Table 3: Means and Standard Deviations of Collagen expression in Figure 4B

|  | Control | PDGF | PDGF+Trep-6 | PDGF+Trep-8 |
| --- | --- | --- | --- | --- |
| Mean*1000 | 0.21 | 0.31 | 0.22 | 0.29 |
| Standard Deviation*1000 | 0.04 | 0.04 | 0.03 | 0.03 |

Supplementary Table 4: Means and Standard Deviations of Fibronectin expression in Figure 5B

|  | Control | PDGF | PDGF+Trep-6 | PDGF+Trep-8 |
| --- | --- | --- | --- | --- |
| Mean*1000 | 0.0006 | 0.0020 | 0.0011 | 0.0010 |
| Standard Deviation*1000 | 0.0001 | 0.0005 | 0.0002 | 0.0003 |

Supplementary Table 5: p-values of all comparisons of expression in Figures 2B, 2D, 4B, 5B

|  | Overall ANOVA | Control vs. PDGF | PDGF vs. PDGF+Trep-6 | PDGF vs. PDGF+Trep-8 |
| --- | --- | --- | --- | --- |
| Figure 2B | <0.001 | <0.001 | <0.001 | <0.001 |
| Figure 2D | <0.001 | 0.935 | <0.001 | 0.537 |
| Figure 4B | <0.001 | <0.001 | <0.001 | 0.285 |
| Figure 5B | <0.001 | <0.001 | <0.001 | <0.001 |
